# Autosomal recessive congenital cataract linked to *EPHA2* in a consanguineous Pakistani family

**Published:** 2010-03-24

**Authors:** Haiba Kaul, S. Amer Riazuddin, Mariam Shahid, Samra Kousar, Nadeem H. Butt, Ahmad U. Zafar, Shaheen N. Khan, Tayyab Husnain, Javed Akram, J. Fielding Hejtmancik, Sheikh Riazuddin

**Affiliations:** 1 National Centre of Excellence in Molecular Biology, University of the Punjab, Lahore, Pakistan; 2 Allama Iqbal Medical College, University of Health Sciences, Lahore, Pakistan; 3 Ophthalmic Genetics and Visual Function Branch, National Eye Institute, National Institutes of Health, Bethesda, MD

## Abstract

**Purpose:**

To investigate the genetic basis of autosomal recessive congenital cataracts in a consanguineous Pakistani family.

**Methods:**

All affected individuals underwent a detailed ophthalmological and clinical examination. Blood samples were collected and genomic DNAs were extracted. A genome-wide scan was performed with polymorphic microsatellite markers. Logarithm of odds (LOD) scores were calculated, and Eph-receptor type-A2 (*EPHA2*), residing in the critical interval, was sequenced bidirectionally.

**Results:**

The clinical and ophthalmological examinations suggested that all affected individuals have nuclear cataracts. Genome-wide linkage analyses localized the critical interval to a 20.78 cM (15.08 Mb) interval on chromosome 1p, with a maximum two-point LOD score of 5.21 at θ=0. Sequencing of *EPHA2* residing in the critical interval identified a missense mutation: c.2353G>A, which results in an alanine to threonine substitution (p.A785T).

**Conclusions:**

Here, we report for the first time a missense mutation in *EPHA2* associated with autosomal recessive congenital cataracts.

## Introduction

Congenital cataracts are one of the major causes of vision loss in children worldwide and are responsible for about one-third of blindness in infants [1,2]. Congenital cataracts can occur in an isolated fashion or as one component of a syndrome affecting multiple tissues. Nonsyndromic congenital cataracts have an estimated frequency of 1–6 per 10,000 live births [3]. They vary markedly in severity and morphology, affecting the nuclear, cortical, polar, or subcapsular parts of the lens or in severe cases the entire lens.

Nearly, one-third of congenital cataract cases are familial [4]. Genes that have been previously reported to be associated with autosomal dominant congenital cataract include crystallins, connexins, beaded filament structural proteins, aquaporin 0, and developmental and transcription factors. Conversely, fewer autosomal recessive cataract loci have been mapped. To date, 12 loci residing on chromosomes 1p34.3-p32.2, 1q21.1, 3p22–24.2, 6p23–24, 9q13–22, 16q21–22, 19q13, 19q13.4, 20p12.1, 21q22.3, 22q11, and 22q12.1 have been mapped, with six of these also causing autosomal dominant cataracts [5-16]. Of these loci, mutations in connexin50 (*GJA8*), glucosaminyl (N-acetyl) transferase 2 (*GCNT2*), heat-shock transcription factor 4 (*HSF4*), lens intrinsic membrane protein (*LIM2*), beaded filament structural protein 1 (*BFSP1*), alphaA-crystallin (*CRYαA*), betaB1-crystallin (*CRYβB1*), and betaB3-crystallin (*CRYβB3*) have been found [6,8,10,12-16].

EPHA2 belongs to the tyrosine kinase family of proteins and is an epithelial cell kinase that has been associated with autosomal dominant cataracts and recently it was implicated in age-related cortical cataracts in humans and mice [17,18]. *EPHA2* belongs to the tyrosine kinase family, and EPHA2 is epithelial cell kinase that interacts with membrane-bound ephrin ligands, which play an important role in morphogenesis and in numerous developmental processes [19]. Structurally, these proteins contain a ligand-binding domain, epidermal growth factor-like domain, and tyrosine kinase catalytic domain [20].

Here we report a consanguineous Pakistani family (PKCC118) with four affected members with nuclear cataracts. Genome-wide linkage analyses localized the disease interval to chromosome 1p. Sequencing of *EPHA2* identified a missense mutation that segregated with the disease phenotype in the family. To the best of our knowledge this is the first report associating mutations in *EPHA2* with autosomal recessive congenital cataracts.

## Methods

### Clinical ascertainment

A total of 100 consanguineous Pakistani families with nonsyndromic cataract were recruited to participate in a collaborative study between the National Centre of Excellence in Molecular Biology, Lahore, Pakistan, and the National Eye Institute, Bethesda, MD, to identify new disease loci causing inherited visual diseases. Institutional Review Board (IRB) approval was obtained from the National Centre of Excellence in Molecular Biology and the National Eye Institute. The participating subjects gave informed consent consistent with the tenets of the Declaration of Helsinki. A detailed medical history was obtained by interviewing family members. Ophthalmic examinations were conducted with slit-lamp microscopy. Blood was drawn by venipuncture with the help of a syringe that is usually attached with a butterfly. Approximately 10 ml of blood samples were drawn from affected and unaffected members of the family and stored in 50 ml Sterilin® falcon tubes (BD Biosciences, San Jose, CA) containing 400 μl of 0.5 M EDTA. Blood samples were kept at −20 °C for long- term storage.

### DNA extraction

DNA was extracted by a nonorganic method, as described by Grimberg et al. [21] with minor modifications. Briefly, aliquots of 10 ml blood samples were mixed with 35 ml of TE buffer (10 mM Tris-HCl, 2mM EDTA, pH 8.0), and the TE-blood mixture was centrifuged at 3,000 rpm (1,800× g) for 20 min. The supernatant was discarded, and the pellet was resuspended in 35 ml of TE buffer and centrifuged at 3,000 rpm (1,800× g) for 20 min. The TE washing was repeated two to three times, and the washed pellet was resuspended in 2 ml of TE. Protein digestion cocktail (6.25 ml; 50 μl [10 mg/ml] of proteinase K, 6 ml TNE buffer [10 mM Tris HCl, 2 mM EDTA, 400 mM NaCl] and 200 μl of 10% sodium dodecyl sulfate) was added to the resuspended pellets and incubated overnight in a shaker (250 rpm) at 37 °C. The digested proteins were precipitated by adding 1 ml of 5 M NaCl, followed by vigorous shaking and chilling on ice for 15 min. The precipitated proteins were pelleted by centrifugation at 3,000 rpm for 20 min and removed. The supernatant was mixed with equal volumes of phenol/chloroform/isoamyl alcohol (25:24:1), and the aqueous layer containing the genomic DNA was carefully collected. The DNA was precipitated with isopropanol and pelleted by centrifugation at 4,000 rpm for 15 min. The DNA pellets were washed with 70% ethanol and dissolved in TE buffer. The DNA concentration was determined with a SmartSpec plus Bio-Rad Spectrophotometer (Bio-Rad, Hercules, CA).

### Genotype analysis

A genome-wide scan was performed with 382 highly polymorphic fluorescent markers from the ABI PRISM Linkage Mapping Set MD-10 (Applied Biosystems, Foster City, CA), having an average spacing of 10 cM. Multiplex PCR was completed in a GeneAmp PCR System 9700 thermocycler (Applied Biosystems). Briefly, each reaction was performed in a 5 μl mixture containing 40 ng genomic DNA, various combinations of 10 mM dye-labeled primer pairs, 0.5 ml 10× GeneAmp PCR Buffer (Applied Biosystems), 1 mM dNTP mix, 2.5 mM MgCl_2_, and 0.2 U Taq DNA polymerase (Applied Biosystems). Initial denaturation was performed for 5 min at 95 °C, followed by 10 cycles of 15 s at 94 °C, 15 s at 55 °C, and 30 s at 72 °C and then 20 cycles of 15 s at 89 °C, 15 s at 55 °C, and 30 s at 72 °C. The final extension was performed for 10 min at 72 °C. PCR products from each DNA sample were pooled and mixed with a loading cocktail containing HD-400 size standards (Applied Biosystems). The resulting PCR products were separated in an ABI 3100 DNA Analyzer (Applied Biosystems) and genotypes were assigned with GeneMapper software (Applied Biosystems).

### Linkage analysis

Two-point linkage analyses were performed using the FASTLINK version of MLINK from the LINKAGE Program Package (provided in the public domain by the Human Genome Mapping Project Resources Centre, Cambridge, UK) [22,23]. Maximum LOD (logarithm of odds) scores were calculated using ILINK from the Linkage Program Package. Autosomal recessive cataract was analyzed as a fully penetrant trait with an affected allele frequency of 0.001. The marker order and distances between the markers were obtained from the Marshfield database and the NCBI chromosome 1 sequence maps. For the initial genome scan, equal allele frequencies were assumed, while for fine mapping, allele frequencies were estimated from 96 unrelated and unaffected individuals from the Punjab province of Pakistan.

### Mutation screening

Primer pairs for individual exons were designed using the primer3 program. The sequences and annealing temperatures are given in Table 1. Amplifications were performed in a 25 μl reaction volume containing 50 ng of genomic DNA, 400 nM of each primer, 250 μM of dNTPs, 2.5 mM MgCl_2_, and 0.2 U *Taq* DNA polymerase in the standard PCR buffer provided by the manufacturer (Applied Biosystems). PCR amplification consisted of a denaturation step at 96 °C for 5 min followed by 40 cycles, each consisting of 96 °C for 30 s followed by 57 °C (or primer set-specific annealing temperature; see Table 1) for 30 s and 72 °C for 1 min. PCR products were analyzed on a 2% agarose gel and purified by by ethanol precipitation. The PCR primers for each exon were used for bidirectional sequencing using BigDye Terminator Ready reaction mix (Applied Biosystems), according to the manufacturer’s instructions. Sequencing products were precipitated and resuspended in 10 μl of formamide (Applied Biosystems) and denatured at 95 °C for 5 min. Sequencing was performed on an ABI PRISM 3100 Automated Sequencer (Applied Biosystems). Sequencing results were assembled with ABI PRISM sequencing analysis software version 3.7 and analyzed with SeqScape software (Applied Biosystems).

**Table 1:** Primer sequences and annealing temperatures of *EPHA2*.

**Exon number**	**Forward**	**Reverse**	**Product Size**	**Annealing Temperature (°C)**
Exon 01	CCAAGGTCCTCCTCCAAAC	GACACCAGGTAGGTTCCAAAG	468	57
Exon 02	TTGGATATGGTGACCCTGTG	TCTGAGCCTGGTGTGAGAAG	372	57
Exon 03a	CTCAGGCCTCAGTTTCCTTC	CTCCTCCACGTTCAGCTTC	484	57
Exon 03b	GCTCCTGCAAGGAGACTTTC	CCAAGATTCCATGATTCCAA	569	56
Exon 04	CACAAGACATTTTGCCGATG	CACGGCTGTGAGGTAGTGTG	433	57
Exon 05	CACACGTGAGTCTTGCAGTG	CCTCCTTAAGCCCCACCT	487	57
Exon 06	AAAGAATCTGGGCTGTGGAG	AGGACGCCATGTCTTCTCTC	427	57
Exon 07	CTAACAAAGGCAAGCCACCT	GAGAAAGGGGCATTTCTAAGTT	369	57
Exon 08	AGTACCCTCTGGAGCCTTCC	CAGGCACTTCGCTACACACT	400	57
Exon 09	TCACTTCCTCCCTGTTCCTC	AGACTTGGACCAGGCTGTG	416	57
Exon 10	AGCCTGGTCCAAGTCTCTGA	TACACCTCCCCAAACTCTCC	365	57
Exon 11	GTGTCACTCGGCAGAAGGT	GTAGAGGAGGTGGGTGCAG	458	57
Exon 12	AGCTTTCCCCACACCTCTC	GGTCACGGTGCACATAGTTC	444	57
Exon 13	GGACAAGTTCCTTCGGGTAA	TACAGGTGTTCTGCCTCCTG	433	57
Exon 14	CAGGAGGCAGAACACCTGTA	TGGAGCAAGCCTAAGAAGGT	377	56
Exon 15	TCCTGTCTGTTTCTGGGATG	GCCATCGTGTCCAGTCTAAG	472	56
Exon 16	CCTGTTGCCCAGATAAGGAG	AGTTCTGCCCTTCTCTTCCA	441	56
Exon 17a	AGCTCTCTTGCCCTACAGGT	GCTAAGTGCTCAGCTGTGTG	499	56
Exon 17b	GGCCACTGGGGACTTTATT	GAAGGCACTAGAGGGACAGG	403	56
Exon 17c	GGTACCTCAAGCCCCATTT	CGGTTTGAATCATCTGCAAC	492	56
Exon 17d	GGGTGTCAAACATTCGTGAG	ACTCTGAGCAGCCTGGAGAT	483	56

### Prediction analysis

The degree of evolutionary conservation of amino acid at positions of interest and their possible impact on the structure of the EPHA2 protein was examined with SIFT and PolyPhen tools available online. Evolutionary conservation of the mutated amino acid (A785) in other EPHA2 orthologs was examined using the UCSC genome browser.

## Results

**Figure 1:**
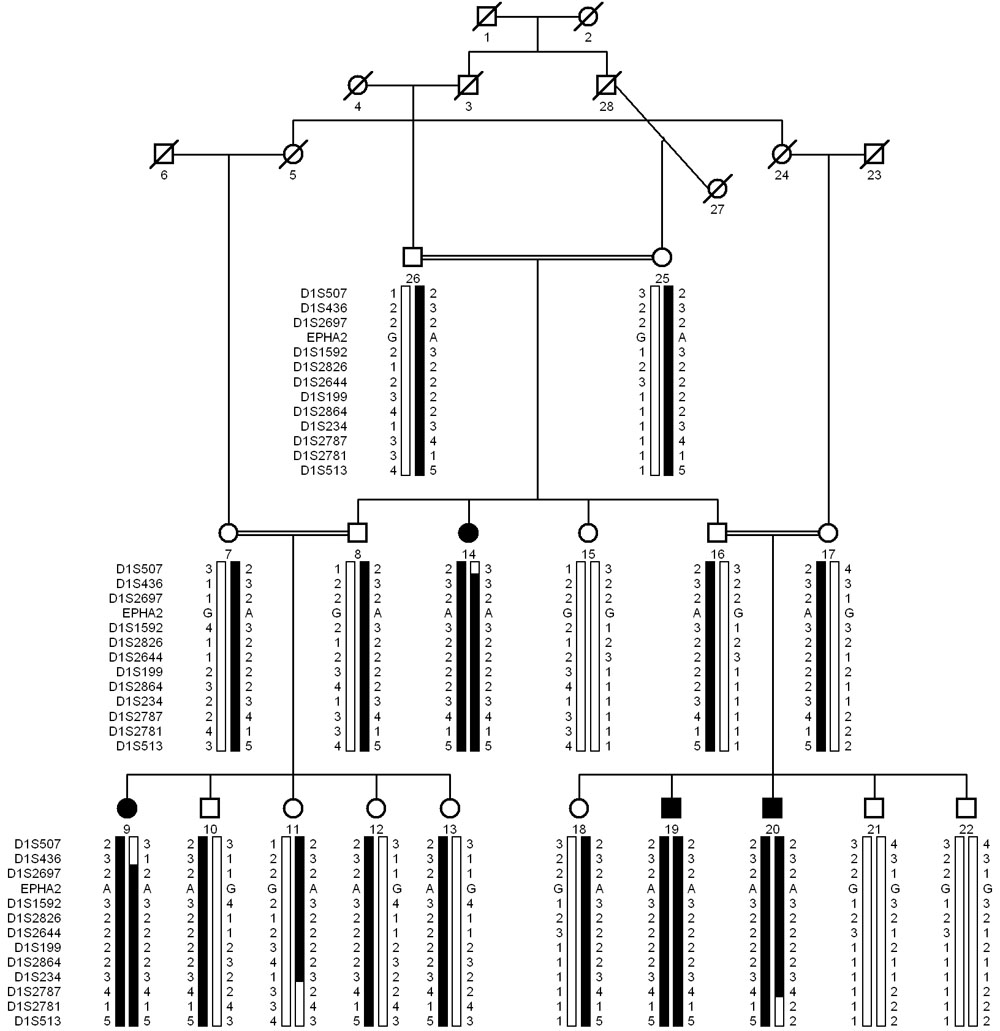
Pedigree drawing and haplotypes of chromosome 1p markers of family PKCC118. Squares are males, circles are females, and filled symbols are affected individuals; the double line between individuals indicates consanguinity and the diagonal line through a symbol is a deceased family member. The haplotypes of 12 adjacent chromosome 1p microsatellite markers are shown; alleles forming the risk haplotype are shaded black, while alleles not co-segregating with cataract are shown in white.

**Figure 2:**
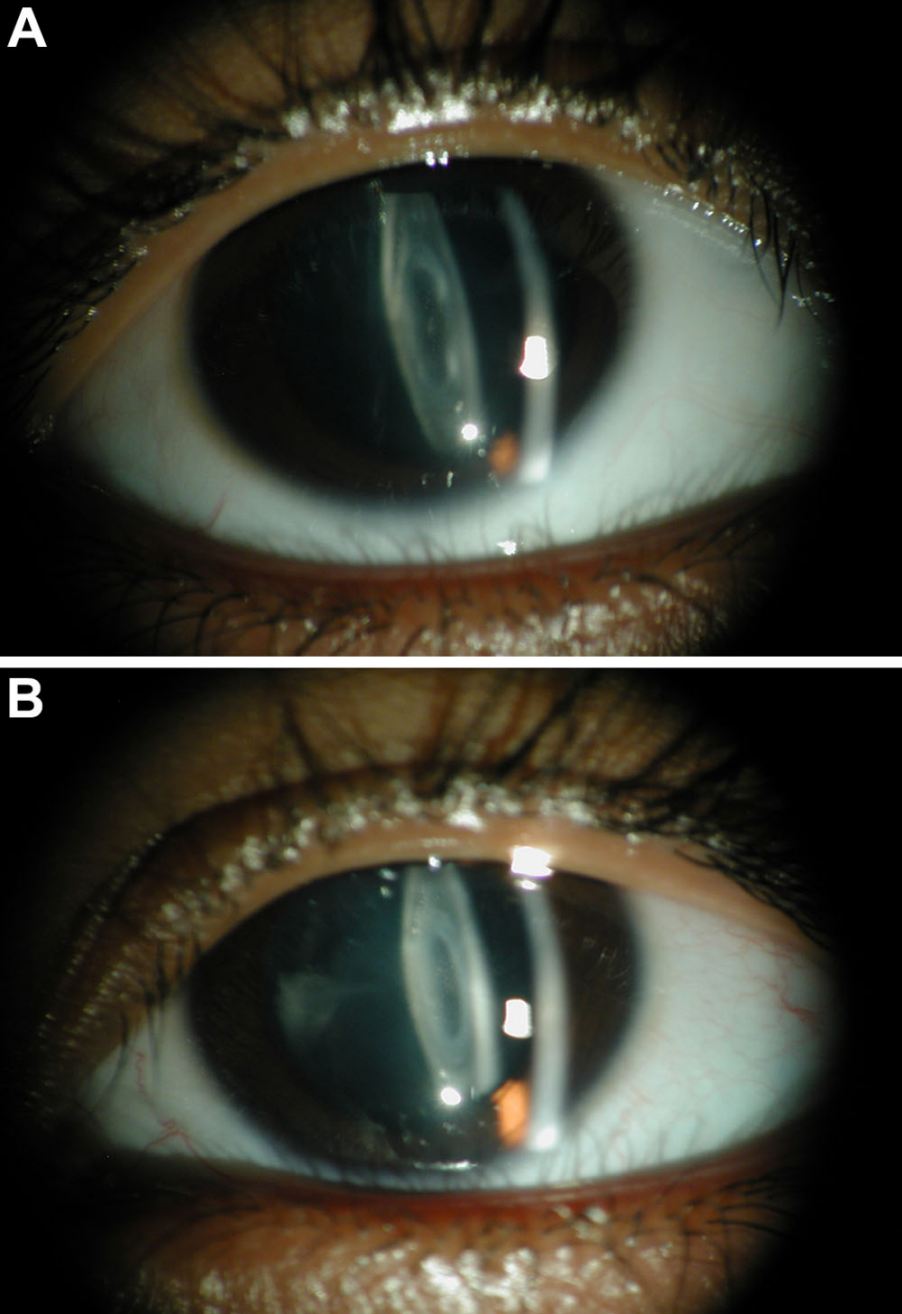
Slit lamp photographs of the affected individuals of family PKCC118. **A**: Individual 19 and **B**: individual 20 of PKCC118 reveal nuclear cataracts that developed in the early years of their lives.

A large consanguineous family, PKCC118, consisting of four affected and 14 unaffected individuals in three consanguineous marriages, was recruited from the Punjab province of Pakistan (Figure 1). A detailed medical history was obtained from all the affected and unaffected individuals of the family, which revealed that cataracts in affected individuals developed in the early years of their life. Clinical examination conducted with slit-lamp microscopy revealed nuclear cataracts (Figure 2). No other ocular or systemic abnormalities were present in the family.

Initially, all the known loci and genes for recessive cataract were excluded by haplotype analysis using closely spaced short tandem repeat (STR) markers (data not shown). Subsequently, a genome-wide scan was completed on a subset of family members and LOD scores of 2.36, 3.23, 4.74, and 1.8 at θ=0 were obtained with D1S2697, D1S199, D1S234, and D12S336, respectively. Additional STR markers from the Marshfield database were genotyped for all the participating members of the family, and this excluded the region at chromosome 12 with a LOD score of **-∞** for D12S99 and D12S1697 (data not shown). Fine mapping with D1S1592, D1S2826, D1S2644, D1S2864, and D1S2787 yielded LOD scores of 4.56, 2.37, 3.60, 5.21, and 5.08 at θ=0, respectively (Table 2).

**Table 2:** Two point LOD scores of chromosome 1P markers for family PKCC118.

**Marker**	**cM**	**Mb**	**0.00**	**0.01**	**0.05**	**0.09**	**0.10**	**0.20**	**0.30**	**Z_max_**	**θ_max_**
D1S507	33.75	15.02	-∞	0.81	1.83	1.95	1.94	1.55	0.95	1.95	0.09
D1S436	37.05	15.87	-∞	2.63	2.86	2.69	2.62	1.79	0.88	2.88	0.03
D1S2697*	37.05	16.41	2.36	2.31	2.11	1.91	1.86	1.34	0.84	2.36	0.00
D1S1592	38.51	18.06	4.56	4.46	4.06	3.65	3.54	2.48	1.43	4.56	0.00
D1S2826	41.92	18.43	2.37	2.31	2.04	1.79	1.72	1.11	0.58	2.37	0.00
D1S2644	43.72	19.02	3.60	3.53	3.26	2.97	2.9	2.14	1.33	3.6	0.00
D1S199*	45.33	19.95	3.23	3.16	2.89	2.61	2.54	1.8	1.03	3.23	0.00
D1S2864	50.28	22.87	5.21	5.11	4.69	4.26	4.14	2.99	1.81	5.21	0.00
D1S234*	55.10	25.15	4.74	4.64	4.22	3.79	3.69	2.56	1.41	4.74	0.00
D1S2787	56.48	28.12	5.08	4.98	4.58	4.17	4.06	2.96	1.81	5.08	0.00
D1S2781	57.83	30.95	-∞	1.25	1.67	1.64	1.61	1.22	0.76	1.67	0.05
D1S513	60.01	31.33	-∞	2.78	2.98	2.16	2.10	1.98	1.42	3.10	0.03

The asterisk indicates marker included in genome wide scan.

Visual inspection of the haplotype supports the results and confirms linkage to chromosome 1p (Figure 1). There is proximal recombination in individual 14 at marker D1S507 and individual 9 at marker D1S436. Similarly, there is distal recombination in individual 20 at D1S2781. Taken together, this places the pathogenic mutation in a 20.78 cM (15.08 Mb) interval flanked by markers D1S436 proximally and D1S2781 distally. Alleles for markers D1S2697, D1S1592, D1S2826, D1S2644, D1S199, D1S2864, D1S234, and D1S2787 are homozygous in all affected individuals.

**Figure 3:**
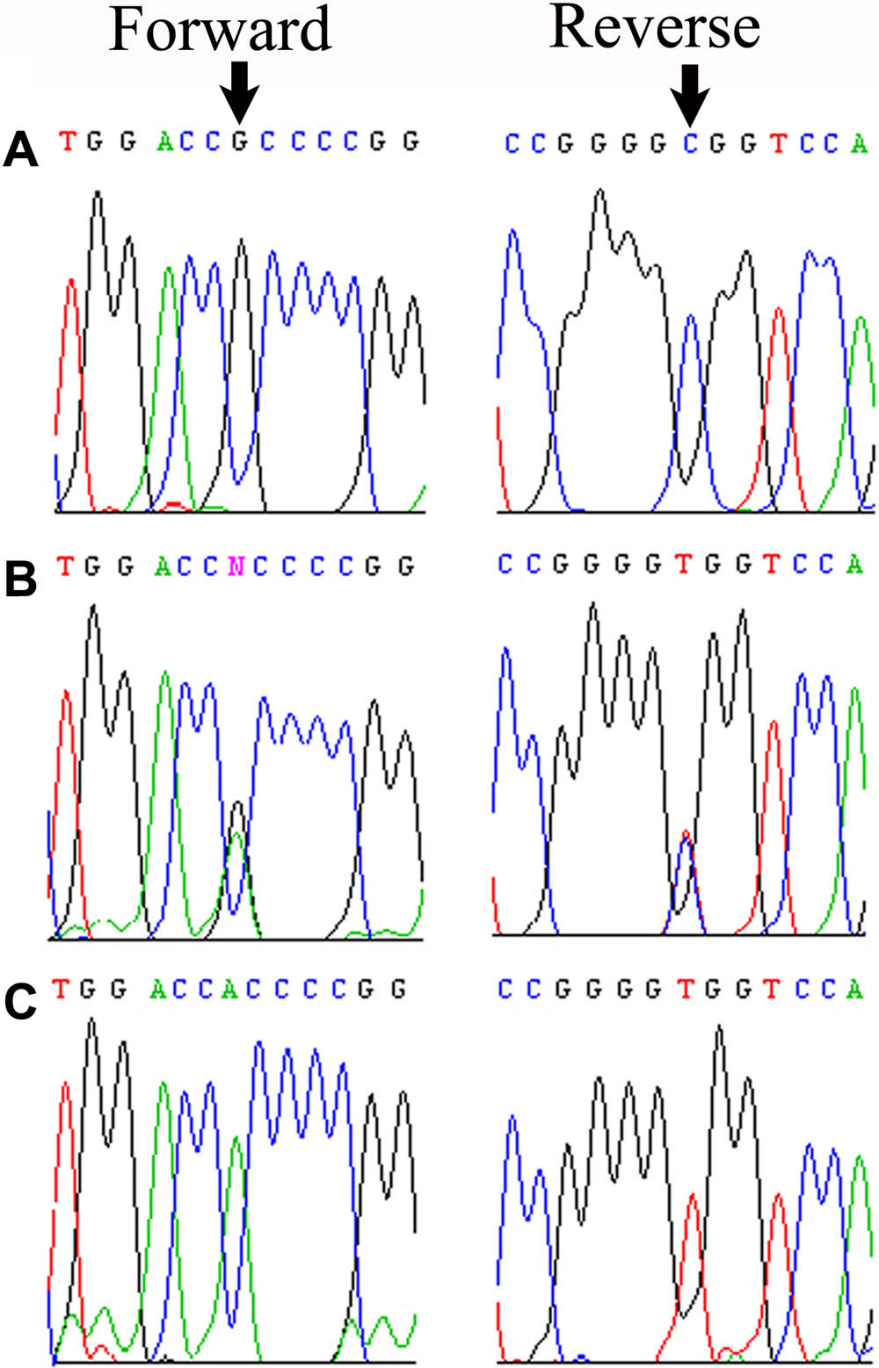
Mutational analyses of Eph-receptor type-A2 in family PKCC118. **A**: Sequence chromatograms of wild type allele in individual 15 showing the translation of alanine. **B**: Individual 10, and **C**: individual 20 are heterozygous and homozygous for c.2353 G>A transition, respectively, which predicts the substitution of an alanine residue for a threonine residue at position 785 (Ala785Thr) in the Eph-receptor type-A2.

Sequencing of *EPHA2* identified a missense mutation: c.2353 G>A that results in a p.A785T, which affects the tyrosine kinase domain of EPHA2. All affected individuals were homozygous for this variation, whereas unaffected individuals were either heterozygous or were homozygous for the wild-type alleles (Figure 3). This variation was not present in 96 ethnically matched control samples. In addition to the pathogenic variation, we identified three single-nucleotide polymorphisms (SNPs) rs2230597, rs3754334, and rs1803527 and a synonymous variation: c.453 C>T (p.T151T).

**Figure 4:**
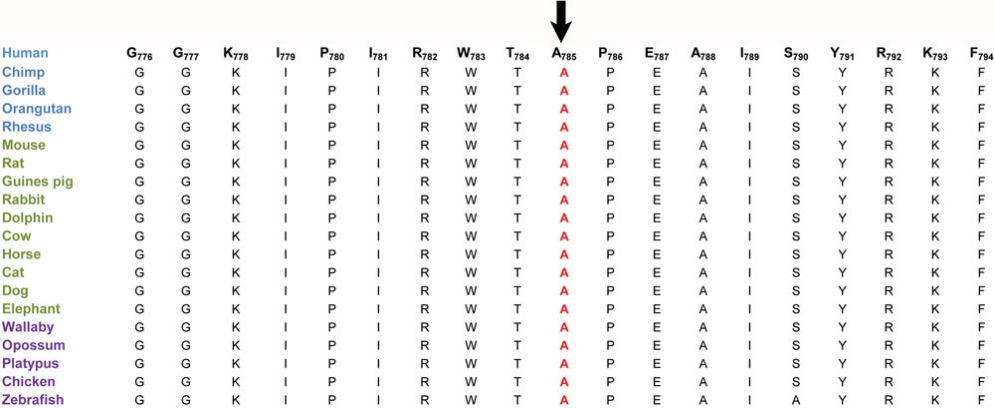
Sequence alignment of amino acids of the Eph-receptor type-A2 illustrating conservation of the amino acid alanine at position 785 and its neighboring amino acids. The organisms shown in blue are primates, in green are placental mammals, and in purple are vertebrates. The arrow points to A785 that is mutated in the family, PKCC118.

To investigate the possible impact of the p.A785T substitution on the EPHA2 protein, we used SIFT and PolyPhen software. SIFT predictions suggested that A785T substitution will not be tolerated by the native three-dimensional structure of the EPHA2 protein. The affected protein function score for A785T was 0.01(amino acids with probabilities <0.05 are predicted to be deleterious). Likewise, position-specific score differences obtained from PolyPhen suggested that A785T substitution could potentially have a deleterious effect on the EPHA2 protein structure. The position-specific independent counts (PSIC) score difference was 2.09 (a PSIC score difference >1.0 is probably damaging). We further analyzed the evolutionary conservation of the substituted amino acids by aligning *EPHA2* orthologues. The results strongly suggest that not only Ala785 but also amino acids in the immediate neighborhood are well conserved among *EPHA2* orthologues (Figure 4).

## Discussion

Here, we report mutations in *EPHA2* associated with autosomal recessive congenital cataracts in a consanguineous Pakistani family. Genome-wide scan localized the critical interval to chromosome 1p with maximum two-point LOD scores of 5.21 at θ=0. Sequencing of *EPHA2* identified a missense mutation that segregates with the disease phenotype in the family. This mutation is predicted to have a deleterious effect on the native structure of the protein and was absent in 96 ethnically matched control samples. Taken together, these results strongly suggest that mutation in *EPHA2* is responsible for recessive congenital cataracts in the Pakistani family.

The linked interval on chromosome 1p36.21-p35.2 was previously mapped in American, British, and Australian families with dominant congenital cataract in three independent reports [24-26]. Recently, Shiels and colleagues [17] reported mutations in *EPHA2* that are responsible for autosomal dominant cataracts. During the preparation of this article, Zhang and colleagues [27] identified mutations in a Chinese and two previously reported British and Australian kindreds, confirming the involvement of *EPHA2* in autosomal dominant cataract.

The human lens is an avascular tissue, where cell-cell junctions are critical for providing nutrient transport and removal of metabolic wastes through intercellular adhesion complexes comprising gap and adherens junctions. Adherens junctions of lens are provided with transmembrane proteins and N-cadherin and β-catenin proteins [28]. Recently, it was demonstrated that ephrin-A5 acts as a regulator for EPHA2, and loss of ephrin-A5 function can lead to cataracts in mice [29]. Further, it has been shown that ephrin-A5 interacts with the EphA2 receptor to regulate the adherens junction complex by enhancing recruitment of β-catenin to N-cadherin [29].

Transparency and precise shape are distinctive features of the lens that are critical for proper light refraction; however, not much is known about the mechanisms that maintain transparency of lens. Elucidating the molecular mechanisms and factors that maintain or disrupt lens transparency is a fundamental precursor for preventing cataract. Identification of new genes associated with cataractogensis will help us better understand the molecular biology of the human lens, including structural and metabolic mechanisms involved in maintaining the clarity of the lens.
